# Pharmacotherapy in Bronchopulmonary Dysplasia: What Is the Evidence?

**DOI:** 10.3389/fped.2022.820259

**Published:** 2022-03-09

**Authors:** Rishika P. Sakaria, Ramasubbareddy Dhanireddy

**Affiliations:** ^1^Department of Pediatrics, University of Tennessee Health Science Center, Memphis, TN, United States; ^2^Department of Obstetrics and Gynecology, University of Tennessee Health Science Center, Memphis, TN, United States

**Keywords:** bronchopulmonary dysplasia, BPD, chronic lung disease, neonates, preterm, neonatal pharmacology

## Abstract

Bronchopulmonary Dysplasia (BPD) is a multifactorial disease affecting over 35% of extremely preterm infants born each year. Despite the advances made in understanding the pathogenesis of this disease over the last five decades, BPD remains one of the major causes of morbidity and mortality in this population, and the incidence of the disease increases with decreasing gestational age. As inflammation is one of the key drivers in the pathogenesis, it has been targeted by majority of pharmacological and non-pharmacological methods to prevent BPD. Most extremely premature infants receive a myriad of medications during their stay in the neonatal intensive care unit in an effort to prevent or manage BPD, with corticosteroids, caffeine, and diuretics being the most commonly used medications. However, there is no consensus regarding their use and benefits in this population. This review summarizes the available literature regarding these medications and aims to provide neonatologists and neonatal providers with evidence-based recommendations.

## Introduction

Northway et al. (1) were the first to describe bronchopulmonary dysplasia (BPD). They coined the term “BPD” to define oxygen and mechanical ventilation (MV) induced injury and the superimposed healing occurring in the lungs of infants with respiratory distress syndrome (RDS) (1). The successful introduction of exogenous surfactant therapy in 1980s resulted in a change from “old BPD” consisting predominantly of airway injury, inflammation and parenchymal fibrosis to “new BPD” characterized by decreased fibrosis, lesser airway injury and more uniform inflation with alveolar simplification. Pulmonary microvascular development is also reduced in some patients with “new BPD” (2, 3). Therefore, BPD was redefined as lung injury in preterm infants requiring ≥28 days of supplemental oxygen (National Institute of Child Health and Human Development (NICHD)/National Heart, Lung and Blood Institute (NHLBI) workshop, 2000). Depending on the amount of supplemental oxygen required at 36 weeks' postmenstrual age (PMA) [for infants < 32 weeks' gestational age (GA)] or at >28 days (for infants ≥ 32 weeks' GA), BPD was classified as mild, moderate, or severe (2).

The advances in treatment methods and the more frequent use of low-flow nasal cannulas with 100% oxygen warranted a newer definition of BPD not dependent solely on supplemental oxygen requirement. To address this, a newer definition of BPD was formulated at the workshop held by NICHD in 2016. This workshop defined BPD as ‘persistent parenchymal lung disease in infants <32 weeks' GA with confirmation of parenchymal disease on chest radiograph and respiratory support requirement for >2 consecutive days at 36 weeks' PMA to maintain transcutaneous oxygen saturation between 90-95%. Furthermore, BPD severity was classified into grades I to III based on the amount of respiratory support, and a class III(A) was defined to include the infants < 36 weeks' PMA, who died because of respiratory failure associated with parenchymal lung disease (4).

In 2019, Jensen et al. concluded that the severity of BPD defined based on the mode of oxygen supplementation at 36 weeks' PMA was the best predictor of morbidity and mortality. Based on a study including 2677 infants (<32 weeks' GA) from 18 centers, the optimal BPD classification at 36 weeks' PMA was defined as: no BPD = no oxygen support, grade 1 = low-flow nasal cannula (≤ 2 L/min), grade 2 = high-flow nasal cannula (>2L/min) or non-invasive positive pressure ventilation (NIPPV), and grade 3 = invasive mechanical ventilation (5).

BPD is a multifactorial disease. Each year, over 10,000–18,000 infants <28 weeks' GA (~ 35%) born in the United States (US) are diagnosed with BPD (6, 7). The risk of BPD increases with decreasing GA and birth weight (5). With more premature infants surviving, the rate of BPD has increased (8). Numerous non-pharmacological and pharmacological interventions have been investigated to prevent and manage BPD and several reviews have been published reviewing the drugs used in BPD. However, there remains no clear consensus on the use of various medications for BPD (9–19). In this review, we summarize the available evidence for the different pharmacological agents currently in use to prevent and manage BPD and aim to provide neonatologists and neonatal practitioners with evidence-based recommendations. Table 1 provides a summary of the drugs commonly used in BPD.

**Table 1:** Summary of drugs used in infants with BPD.

**Commonly used drugs**	**Proposed mechanism of action**	**Our recommendations regarding use**
**Antenatal corticosteroids** • IM Dexamethasone (6 mg q12h x 4 doses)(13) • IM Betamethasone (12 mg q24h x 2 doses)(13)	• ↑ alveolar differentiation into type II pneumocytes • ↑ surfactant production • Improved pulmonary blood flow	•No evidence regarding improved BPD outcomes but improves survival. • Should be given to all eligible mothers per ACOG guidelines^†^
**Surfactant** [Intratracheal administration; all dosages based on phospholipids/kg of birth weight] • Poractant alfa/Curosurf ® (1^st^ dose: 200 mg/kg; 2^nd^/3^rd^ doses: 100 mg/kg/dose 12 hours apart, if needed 12 hours after the 1^st^ dose)^‡^ • Calfactant/Infasurf ® 105 mg/kg at least 12 hours apart as needed for maximum 3 doses)^§^ • Beractant/Survanta ® (100 mg/kg/dose at least 6 hours apart as needed for maximum 4 doses)^£^	• ↓ alveolar surface tension • ↑ lung compliance • ↑ functional residual capacity • ↓ mechanical ventilation induced lung injury	•No evidence regarding improved BPD outcomes, but improves survival in infants with RDS. • INSURE/LISA should be used for surfactant administration in spontaneously breathing infants. • Aerosolized surfactant/combined administration of intratracheal surfactant and corticosteroid should be reserved for research setting.
**Methylxanthines** • Caffeine citrate (loading dose: 10-20 mg/kg maintenance dose: 5 mg/kg/day)	• Prevention of apnea of prematurity through ◦ CNS stimulation through antagonism of adenosine receptors ◦ Improved diaphragmatic contractility ◦ ↑ central and peripheral chemoreceptors' responsiveness to CO_2._ • Improved minute ventilation • ↓ inflammation • Prostaglandin inhibition	• Early caffeine administration (as soon as possible after birth) recommended for all infants ≤30 weeks gestation, to be continued until PMA 34-36 weeks.
**Postnatal corticosteroids** • Systemic: ◦ Early (<8 days): ▪ Hydrocortisone: (IV 0.5 mg/kg/dose- q12h x 7 days followed by q24h x 3 days) (73) ◦ Late (>7 days): ▪ Dexamethasone: (IV/PO: 0.075 mg/kg q12h x 3 days, 0.05 mg/kg q12h x 3 days, 0.025 mg/kg q12h x 2 days, and 0.01 mg/kg q12h x 2 days). (75)	• ↓ inflammation	• Early dexamethasone use is not recommended. • Early low-dose hydrocortisone may be considered in centers with high baseline BPD incidence. • Late systemic corticosteroids may be used after discussing with parents between 2-7 weeks of life, in select patients when benefit is likely to outweigh the risks.
• Inhaled: ◦ Budesonide ◦ Fluticasone ◦ Beclomethasone		• Early use not recommended due to increased risk of mortality. • No evidence regarding benefit in prevention/management of BPD
**NSAIDs** • Indomethacin	• PDA closure through inhibition of prostaglandin E2 (PGE2)	• Prophylactic use not recommended
Vitamin A (IM 5000 IU/dose 3 times per week x 12 doses) (108)	• ↑ mean alveolar area • ↑ alveolar regeneration • ↓ fibrosis and oxidative damage	• May be used for prophylaxis in centers with a high baseline incidence of BPD
**Diuretics** • Loop diuretics: ◦ Furosemide (1 mg/kg/dose) • Thiazides ◦ Chlorothiazide ◦ Hydrochlorothiazide • Potassium sparing diuretics ◦ Spironolactone	• ↑ lung compliance • ↑ tidal volume • ↓ airway resistance (+/−) • ↓ pulmonary edema	• Sporadic doses of furosemide may be used for acute respiratory decompensation with signs of pulmonary edema. • Long term use not recommended.
**Inhaled bronchodilators** • β-agonists	• ↓ airway resistance • ↑ lung compliance	• May be trialed for patients with signs of increased airway resistance. • May worsen tracheomalacia if present. • MDI with spacer preferred over nebulizer for delivery.
◦ Isoproterenol ◦ Albuterol/salbutamol ◦ Levalbuterol ◦ Terbutaline		
• Anticholinergics ◦ Ipratropium bromide ◦ Atropine		
**PDE-5 inhibitors** • Sildenafil	• Smooth muscle relaxation and pulmonary vasodilatation • Improved alveolarization • ↑ angiogenesis • ↓ inflammation/fibrosis	• May be used when evidence of BPD-PH exists on echocardiography or cardiac catheterization, in consultation with pediatric cardiologist and pediatric pulmonologist.
**Inhaled pulmonary vasodilators** • iNO	• Pulmonary vasodilatation • ↑ lung volume and dynamic compliance by facilitation of SP-B induced decrease in surface tension. • ↓ inflammation/fibrosis • ↓ endothelial cell apoptosis	• May be used during acute PH crises • Routine use of BPD-PH or for BPD prophylaxis not recommended.
**ET-1 receptor antagonists** • Bosentan	• Reversal of endothelin-mediated smooth muscle constriction, hypertrophy and hyperplasia.	• Routine use not recommended. • Use as a second-line agent for BPD-PH must be in consultation with a pediatric cardiologist and pediatric pulmonologist and after discussion with parents.
**Macrolide antibiotics** • Azithromycin • Clarithromycin • Erythromycin	• Anti-microbial activity against Ureaplasma • ↓ inflammation	• May be used in research setting only

† *Antenatal corticosteroids (ACS) recommended for all pregnant women between 24 0/7 to 33 6/7 weeks of gestation at risk of delivery within seven days, for pregnant women at risk of delivery between 23 0/7 to 23 6/7 weeks of gestation, if resuscitation is desired and for women between 34 0/7 to 36 6/7 weeks of gestation with a risk of preterm delivery within seven days who have not previously received ACS*.

‡ *Curosurf. Available online at: https://resources.chiesiusa.com/Curosurf/CUROSURF_Users_Guide.pdf (2019). (accessed November 15, 2021)*.

§ *Infasurf. Available online at: https://infasurf.com/prescribing-information/ (2018). (accessed November 15, 2021)*.

£ *Survanta. Available online at: https://www.rxabbvie.com/pdf/survanta_pi.pdf (2020). (accessed November 15, 2021)*.

## Medications Used in BPD

### Antenatal Corticosteroids

In 1972, Liggins et al. first showed that antenatal corticosteroids (ACS) promoted lung maturation and prevented RDS in preterm infants (20). About two decades later, the National Institute of Health (NIH) consensus panel recommended ACS use for all impending preterm births 24–34 weeks of gestation (21). ACS promote alveolar epithelial cell differentiation into type II pneumocytes and increase surfactant protein (SP)-A and SP-B expression and the overall surfactant production. They also improve pulmonary blood flow via endothelial nitric oxide synthase activation and increase epithelial Na channels, thus improving respiratory function (22–25).

The most common regimens for ACS administration used throughout the world include betamethasone and dexamethasone. The American College of Obstetrics and Gynecology (ACOG) recommends two doses of intramuscular (IM) betamethasone or four doses of IM dexamethasone for all pregnant women between 24 0/7 to 33 6/7 weeks of gestation and at risk of delivery within seven days (26). For pregnant women at risk of delivery between 23 0/7 to 23 6/7 weeks of gestation, ACS should be administered if resuscitation is desired. ACOG also recommends ACS for women between 34 0/7 to 36 6/7 weeks of gestation with a risk of preterm delivery within seven days who have not previously received ACS (26).

A Cochrane review comparing various ACS regimens found that dexamethasone was associated with lower rates of intraventricular hemorrhage (IVH) compared to betamethasone. Neonatal death, RDS, or neurodevelopmental outcome at 18 months were similar with the two drugs (27). Despite a clear improvement in mortality and RDS, the 2020 Cochrane review comparing ACS with placebo noted no significant difference in the incidence of BPD between the two groups (28). Another meta-analysis including infants <25 weeks' GA from nine observational studies showed that the incidence of BPD was higher with ACS exposure (Odds Ratio (OR):1.32; 95% confidence interval (CI): 1.04–1.67); however, this is more likely due to the significant decline in mortality (OR: 0.48; 95% CI: 0.42–0.55) as well as the combined outcome of BPD and death (OR: 0.58; 95% CI: 0.42–0.79) with ACS use. (29).

*Given the improvement in the incidence of RDS and mortality, in the absence of contraindications, we recommend routine administration of ACS for all pregnant women based on ACOG criteria*.

### Surfactant

Fujiwara et al. were the first to describe successful exogenous surfactant replacement therapy in preterm infants with RDS in 1980 (30). Several multicenter studies on surfactant use followed, leading to the FDA approval of surfactant in premature infants in 1990. Since then, surfactant is routinely used in neonates with RDS. Surfactants available for clinical use include synthetic and animal-derived surfactants, with animal-derived surfactants being the most commonly used. Surfactant improves lung compliance and increases the functional capacity by decreasing alveolar surface tension and preventing alveolar collapse (31). In addition to reducing the severity of RDS, a Cochrane review in 1998 showed that intratracheal administration of surfactant improved mortality, air-leak syndromes, and chronic lung disease (CLD) (32). However, there have been marked improvements in ventilation strategies since this review, including the introduction of non-invasive positive pressure ventilation (NIPPV), and the benefit of surfactant in BPD prevention was not shown in later studies.

The SUPPORT trial published in 2010 was a multicenter randomized controlled trial (RCT). It consisted of 1316 infants of 24 0/7 to 27 6/7 weeks' GA, randomized to receive continuous positive airway pressure (CPAP) or intubation and surfactant administration within the 1st hour of life. The incidence of BPD at 36 weeks' PMA was similar between the two groups (33).

The role of timing and method of surfactant administration in preventing BPD has also been studied. In a Cochrane meta-analysis, there was a decreased incidence of BPD and air leak syndromes in infants receiving early surfactant followed by early extubation to CPAP compared to those receiving selective surfactant and continued MV (34). Another Cochrane review consisting of six RCTs showed that in intubated infants receiving selective surfactant, the risk of CLD, neonatal mortality, and that of CLD or death at 36 weeks' PMA was lower when surfactant was administered early (within two hours of life) compared to late administration (35).

MV itself is associated with lung injury, and it is known that avoiding MV decreases the incidence of BPD (36). In 1994, Verder et al. introduced the INSURE method to reduce the duration of MV in neonates stable on non-invasive ventilation (37). Over a decade later, to avoid MV, Kribs et al. introduced the “less invasive surfactant administration” (LISA) or the “minimally invasive surfactant therapy” (MIST), where surfactant is delivered intratracheally to an infant on CPAP via a thin catheter (38).

In a meta-analysis by Isayama et al., no statistically significant differences were found in the incidence of CLD, death, or the combined outcome of CLD or death between infants receiving nasal CPAP and those receiving early surfactant using the INSURE (Intubate-SURfactant-Extubate) method (39).

Individual RCTs studying the incidence of BPD have found LISA to be either superior or equivalent to conventional surfactant administration (40–43). A meta-analysis found LISA to be associated with lower BPD incidence at 36 weeks' PMA and the incidence of the composite outcome of death or BPD at 36 weeks' PMA compared to conventional surfactant administration via intubation (44). Similar results were noted in the recent Cochrane review by Abdel-Latif et al. (BPD-relative risk (RR): 0.57; 95% CI: 0.45–0.74; death-RR: 0.63; 95% CI: 0.47–0.84; combined BPD or death-RR: 0.59, 95% CI: 0.48–0.73) (45).

When compared to the various non-invasive ventilation strategies, the meta-analysis by Isayama et al. found LISA to be associated with the lowest likelihood of the composite outcome of death or BPD at 36 weeks' PMA (46). However, the recently published OPTIMIST-A trial, a multicenter masked RCT that compared surfactant treatment using a thin catheter vs. sham treatment in preterm infants 25 to 28 6/7 weeks of gestation on CPAP, found no difference in the incidence of death or the composite outcome of death or BPD at 36 weeks' PMA between the two groups. The incidence of BPD in survivors was lower in the MIST group (47).

Another method of surfactant administration is via laryngeal mask airway (LMA). However, an RCT consisting of 103 infants 28 0/7 to 35 6/7 weeks of gestation requiring 0.3–0.4 fractional inspired oxygen (fiO_2_) showed that BPD incidence at 36 weeks' PMA was similar in the groups receiving either CPAP or surfactant via LMA (48). Aerosolized surfactant has been used to treat RDS; however, no RCTs have studied its utility in preventing BPD.

Some researchers have studied intra-tracheal administration of combined corticosteroid and surfactant for BPD prevention. In a meta-analysis, a lower risk of BPD was noted when corticosteroids (inhaled or intratracheal) and surfactant were administered concurrently (RR 0.58, 95% CI 0.41 to 0.82) compared to surfactant alone (49). The “Budesonide in Babies” (BiB) trial (NCT04545866) by the NICHD study group is a large RCT that will study the efficacy of intratracheal surfactant administered in combination with budesonide compared to surfactant alone for the prevention of BPD and death and is currently enrolling patients (50). Table 2 lists the ongoing RCTs evaluating various drugs for the management of BPD.

**Table 2:** Ongoing randomized controlled clinical trials evaluating pharmacotherapy for BPD prevention/treatment listed on ClinicalTrials.gov/ISRCTN registry.

**Study identifier**	**Drug evaluated in the trial**	**Primary outcome measures**	**Estimated completion date**	**Trial status**
NCT01039285	Exogenous surfactant (Curosurf) (CURDYS trial)	Duration of assisted ventilation	June 2022	Active, not recruiting
NCT04825197	Late surfactant (LATE-REC-SURF trial)	Time to first successful extubation	February 2025	Recruiting
NCT04545866	Budesonide + Surfactant (BiB trial)	Physiologic BPD at 36 weeks' PMA or death	September 2025	Recruiting
NCT04862377	Budesonide + Surfactant (BuS trial)	1. Lung ultrasound score at 7 days of life	April 2026	Not yet recruiting
		2. IL-6 concentration in respiratory secretions at 7 days of life		
NCT03086473	Caffeine	Endotracheal intubation within 12 hours of life	July 2022	Active, not recruiting
NCT01353313	Hydrocortisone	1. Improvement in survival without physiologically defined moderate to severe BPD at 36 weeks' PMA	January 2025	Active, Not recruiting
		2. Survival without moderate or severe neurodevelopmental impairment at 22-26 months corrected age		
NCT02884219	Ibuprofen, Indomethacin (BeNeDuctus Trial)	Composite of mortality, NEC and/or BPD at 36 weeks' PMA	June 2021	Recruiting
ISRCTN84264977	Ibuprofen (Baby-OSCAR Trial)	Composite of mortality or BPD at 36 weeks' PMA	January 2022	Completing, awaiting results.
NCT04563429	Vitamin A	1. Incidence of BPD at 28 days	October 2023	Not yet recruiting
		2. Mortality at 28 days		
NCT03946891	Nebulized furosemide	Daily percent change from pre-treatment FiO2 requirement measurement	June 2023	Recruiting
NCT03576885	iNO	1.Death	December 2021	Recruiting
		2.BPD		
NCT00515281	iNO (NOVA2 trial)	1. Neurodevelopment at 2 years	June 2021	Active, not recruiting
		2. BPD at 36 weeks' PMA		
NCT04447989	Sildenafil (SILDI-SAFE trial)	Safety based on incidence of hypotension	December 2023	Recruiting
NCT03142568	Sildenafil (SIL02)	Safety as determined by adverse event	September 2022	Recruiting
ISRCTN11650227	Azithromycin (AZTEC Trial)	Survival without BPD at 36 weeks' PMA	September 2022	Recruiting
NCT03645525	hUC-MSC	Oxygen requirement 3 days post-transplant	April 2023	Recruiting
NCT01897987	PNEUMOSTEM® (hUC-MSC)	Respiratory outcome: readmission rate and duration of the hospital stay due to respiratory infection (6-60 months corrected age)	Completed	Results pending
NCT03392467	PNEUMOSTEM® (hUC-MSC)	Combined outcome of BPD or death at 36 weeks' PMA	July 2021	Recruiting
NCT04003857	PNEUMOSTEM® (hUC-MSC)	Respiratory outcome: readmission rate and duration of the hospital stay due to respiratory infection (6-60 months corrected age	June 2027	Recruiting
NCT04440670	Autologous cord blood mononuclear cells	Frequency of BPD at 36 weeks' PMA	December 2021	Recruiting
NCT02999373	Autologous cord blood mononuclear cells	Frequency of BPD at 36 weeks' PMA	Completed	Results pending
NCT03253263	SHP607 (Mecasermin Rinfabate)	Time to final weaning off respiratory technology support from day 1 through 12 months corrected age	February 2025	Active, not recruiting
NCT04662151	AT-100 (rhSP-D)	Safety and tolerability of AT-100)	February 2022	Not yet recruiting
NCT04078906	SMOFlipid	1.Incidence of BPD at 36 weeks' PMA	October 2024	Recruiting
		2.Fatty acid profile		
		3. Proinflammatory cytokine response		
		4.Lipid peroxidation measures		

*rhSP-D, recombinant human surfactant protein D*.

*Mecasermin Rinfabate: Complex compound consisting of insulin-like growth factor (IGF)-1 and IGF-binding protein 3 (IGFBP3). (Kemp SF. Mecasermin rinfabate. Drugs Today (Barc). (2007) 43:149–55. doi: 10.1358/dot.2007.43.3.1079876)*.

A double-blind RCT consisting of 118 infants <33 weeks' GA requiring MV on day 14 and a fiO_2_ > 0.3 failed to demonstrate any benefit of late surfactant over placebo in lowering the incidence of severe BPD/death at 36 weeks PMA. However, infants who received surfactant had lower respiratory morbidity before one year of age (51). The TOLSURF study, which included infants ≤ 28 weeks' GA on MV at 7-14 days and inhaled nitric oxide (iNO), yielded similar results (52, 53).

BPD incidence was similar in infants treated with protein-free synthetic surfactant and animal-derived surfactant in a Cochrane review (RR 1.0, 95% CI 0.93–1.07) (54).

*For neonates with RDS, surfactant administration is the standard of care and should not be withheld. In our unit, we administer prophylactic surfactant in the delivery room for all infants* <*27 weeks' GA. However, this practice cannot be routinely recommended, and individual unit practices should be followed. Efforts should be made to wean the infant off MV as soon as possible after surfactant use. In spontaneously breathing infants, LISA or INSURE methods for surfactant administration are preferred to avoid MV-related lung injury. While LISA appears to be a promising new technique, larger RCTs are required to determine its role in preventing BPD. We do not recommend routine aerosolized surfactant use or combination with intratracheal steroids outside the scope of research at this time*.

### Caffeine

Caffeine is widely used in the neonatal intensive care unit (NICU). In 1977, in a prospective study consisting of eighteen patients, Aranda et al. first showed that caffeine was effective for treating apnea of prematurity (55). Caffeine is a methylxanthine class-central nervous system (CNS) stimulant that primarily acts by inhibiting the adenosine receptors A1 and A2A in the brain (56–58). In addition, caffeine improves diaphragmatic contractility and prevents diaphragmatic fatigue by increasing intracellular Ca^2+^ and responsiveness of the central and peripheral chemoreceptors to CO_2_, resulting in increased minute ventilation (58). The bronchodilator effect of caffeine was shown to improve lung mechanics and minute ventilation in sixteen preterm infants with BPD (59). Additionally, caffeine also induces diuresis and natriuresis via antagonism of the renal tubular adenosine A1 receptors (60). However, neither the bronchodilator effect nor the diuretic effect appears to be the primary mechanism of action of caffeine.

The caffeine for apnea of prematurity (CAP) trial was a landmark trial in neonatology. It was a multicenter RCT that studied the long-term effects of caffeine administration starting before ten days of age (median: 3 days) in infants <1,500 g on the combined outcome of death and neurodevelopmental outcomes at 18–21 months of corrected age. This study not only established the safety of long-term caffeine use in premature infants but also showed a decreased incidence of BPD with early caffeine administration (*p* < 0.001) (61). By preventing apnea of prematurity, caffeine reduces the need for intubation and facilitates early successful extubation of ventilated infants, thus decreasing the MV-associated lung injury, which plays a key role in BPD pathogenesis (62). The CAP trial also showed that only 33.8% of infants exposed to caffeine underwent medical or surgical patent ductus arteriosus (PDA) closure compared to 50.7% of infants exposed to placebo (*p* < 0.001) (61). The low rate of PDA requiring treatment in the caffeine group may be related to the prostaglandin antagonist effect of methylxanthines (63). Long-term exposure to moderate-severe PDA has been implicated in the pathogenesis of BPD (64).

Animal studies have also shown that caffeine prevents inflammation, improves alveolarization, and upregulates vascular endothelial growth factor (VEGF) and hypoxia-inducible factor 1α (HIF-1α) expression, thus improving pulmonary vascularization following hyperoxia-induced lung injury (65–68). Although one study showed increased alveolar apoptosis with caffeine administration in mice lung exposed to hyperoxia; this effect could be the result of a higher dose used than in other studies (69).

The benefits of caffeine in BPD prevention also depend on the timing of initiation of therapy. The definition of early administration has changed since the CAP trial and is now defined as caffeine administration before three days. In a retrospective study, early administration (<3 days) of caffeine was associated with a significantly lower rate of BPD and the composite outcome of death or BPD compared to late caffeine administration (≥ 3 days) (70). Several RCTs and meta-analyses have confirmed this finding (71, 72). The National Institute for Health and Care Excellence (NICE) recommends routine caffeine use for all neonates ≤ 30 weeks' GA as early as possible (73). An RCT from the Netherlands consisting of infants between 24–30 weeks' GA showed that the infants who received caffeine immediately after birth in the delivery room had significantly higher tidal volumes, minute ventilation, and rate of rise to maximum tidal volume compared to those who received caffeine later in the NICU (74). However, the sample size in this study was small (*n* = 30) and it did not study the long-term effects, including MV duration, BPD, IVH, sepsis, or NEC (74). Another prospective study (the CAFROOM trial, NCT04044976) that will consist of 40 infants 25 0/7 to 29 6/7 weeks GA receiving an initial dose of caffeine (20 mg/kg) in the delivery room within 10 minutes of life is currently underway (75).

A higher maintenance dose of caffeine was associated with lower rates of BPD than the standard dose in many recent meta-analyses; however, firm recommendations could not be provided due to a low level of evidence (76–78). The follow-up data from 142 children enrolled in the CAP trial at the Royal Women's Hospital, Melbourne (52% treated with caffeine and 48% treated with placebo) showed that the caffeine group had significantly improved expiratory flow rates at 11 years compared to the placebo group (79).

The use of caffeine may be associated with side effects, including hyponatremia, hypertension, hyperglycemia, feeding intolerance, and tachycardia. The risk of necrotizing enterocolitis (NEC) was not increased with caffeine use (80).

*In line with the NICE guidelines, we recommend routine caffeine use for all premature infants* ≤ *30 weeks' GA soon after birth*.

### Postnatal Corticosteroids

Lung inflammation is often seen in BPD and plays a significant role in its pathogenesis. Corticosteroids, both systemic and inhaled, with their anti-inflammatory action, are often used for BPD prevention and management (81). However, despite over 80 RCTs studying their efficacy and safety, the timing and indications for postnatal corticosteroids (PNS) use for BPD remain a topic of debate among neonatologists (82).

#### Systemic Corticosteroids

The first RCT assessing the efficacy of systemic PNS in preterm infants (2–6 weeks age) was conducted in 1985 by Avery et al. The study group infants were weaned off MV within 72 hours of dexamethasone treatment and showed a significant improvement in pulmonary compliance compared to the control group (83).

Dexamethasone and hydrocortisone are the most frequently used systemic PNS. Both early (≤7 days) and late (>8 days) administration has been evaluated in various RCTs. There is a wide variation in published studies regarding the dosing of both dexamethasone and hydrocortisone used for BPD prevention (84, 85).

##### Early Systemic PNS

The Cochrane review by Doyle et al. (85) included 32 RCTs with early PNS use; 21 studies used dexamethasone and 11 studies used hydrocortisone. In this review, the risk of BPD and the combined outcome of BPD or death was lower with early PNS. Although there was no increase in mortality, the risk of gastrointestinal (GI) bleeding increased approximately two times and that of cerebral palsy (CP) increased almost 1.5 times (85). Interestingly, in the subgroup analysis, both the decreased BPD and the increased risks of CP and GI bleeding were seen in the dexamethasone group and not in the hydrocortisone group. In addition, the incidence of hyperglycemia, hypertension, and major neurosensory disability was higher, and that of retinopathy of prematurity (ROP) and PDA was lower with the dexamethasone group (85).

The PREMILOC trial deserves a special mention. Infants <28 weeks' GA were randomized to receive early low-dose intravenous (IV) hydrocortisone (0.5 mg/kg/dose; twice daily for 7 days followed by once daily for 3 days) or a placebo. Hydrocortisone significantly improved BPD-free survival at 36 weeks' PMA (60% vs 51%; *p* = 0.04) (86).

##### Late Systemic PNS

The DART (Dexamethasone A Randomized Trial) study by Doyle et al. is a randomized placebo-controlled trial that enrolled 70 infants (<28 weeks' GA or <1,000 g birth weight) dependent on MV at >1 week of life. The treatment (DART) group received 0.89 mg/kg dexamethasone over 10 days (87). This low-dose dexamethasone facilitated successful extubation in more infants by 10 days of treatment (odds ratio [OR]: 11.2; 95% CI: 3.2–39). However, BPD at 36 weeks' PMA, CP at two years of age, and mortality between the two groups remained unchanged with DART (87, 88).

In a retrospective study including 951 infants <27 weeks' GA, the lowest rate of BPD was noted when infants were treated with PNS between weeks two to seven but higher with treatment from week eight onwards (89).

In the Cochrane review assessing late systemic PNS for BPD prevention (2017), preterm infants were found to have a lower incidence of BPD and that of combined death or BPD at 36' weeks PMA with late PNS use, likely due to the facilitation of extubation. Unlike early PNS, late PNS use did not increase the risk of GI bleeding or CP but was associated with an increased rate of hyperglycemia, ROP, and hypertension. Out of the 21 RCTs in this review, only one used late hydrocortisone, while others used late dexamethasone (84).

The STOP-BPD trial was a double-blinded RCT recently published (2019) in which 372 infants <30 weeks and/or 1,250 g birthweight were randomly assigned to receive systemic hydrocortisone (72.5 mg/kg over 22 days) or a placebo. Infants were enrolled if they were MV-dependent in the 2^nd^ week of life. There was no significant difference in the BPD incidence at 36 weeks' PMA or the composite outcome of death or BPD at 36 weeks' PMA with hydrocortisone use. However, death at 36 weeks' PMA was higher in the placebo group. Despite an initial decrease in extubation failure with hydrocortisone, this difference was not seen 21 days after starting the treatment (90).

Prednisolone and betamethasone are less frequently used for the management of BPD (91–94). Two RCTs compared betamethasone to hydrocortisone and dexamethasone, respectively, and found them comparable (91, 92). In a prospective study, Bhandari et al. demonstrated that prednisolone administration facilitated weaning off supplemental oxygen in infants >36 weeks' PMA. However, multiple courses of prednisolone were not practical (93). In a retrospective study, improvement in respiratory status was observed after one week of prednisolone therapy in mechanically ventilated infants; however, no further improvement was noted with continued therapy after one week (94).

Various studies have demonstrated an increase in the risk of neurodevelopmental impairment (NDI) with both early and late dexamethasone use (95–97). In a meta-analysis by Doyle et al., infants with a low baseline risk of moderate to severe BPD (< 33%) had a higher incidence of both CP and death with PNS use. On the contrary, the composite incidence of death or CP in those with a higher baseline risk (>60%) of BPD is lower with PNS administration (98). A retrospective study by Powell et al. also demonstrated a significant positive correlation between the cumulative dexamethasone dose and CP (99).

Another adverse effect of systemic PNS use is the potential to cause adrenal suppression, which occurs when supraphysiologic doses of corticosteroids (equivalent to > 10 mg/m^2^/day) are administered for >14 days (82).

The policy statement by the American Academy of Pediatrics (AAP) Committee on fetus and newborn issued in 2010 and reaffirmed in 2014 did not recommend the use of high-dose dexamethasone (~0.5 mg/kg per day) and noted that there was insufficient evidence for the use of low-dose dexamethasone (<0.2 mg/kg per day) and hydrocortisone for prevention/management of BPD (100). AAP has not revised this statement since then. A recent article by Stark and Eichenwald recommended use of low-dose dexamethasone in infants who continue require mechanical ventilation and oxygen support. However, this was a weak (grade 2C) recommendation and has not been officially endorsed by the AAP (101).

#### Inhaled Corticosteroids

To overcome the systemic side-effects of corticosteroids, inhaled corticosteroids (ICS) such as budesonide, fluticasone, and beclomethasone have been used to prevent/manage BPD. Both late and early use of ICS in preterm infants has been studied. Despite being commonly used in extremely preterm infants, both before and after discharge from the NICU, the evidence for ICS use remains unclear.

In the NEuroSIS trial, a multinational RCT, high dose (200-400 μg) inhaled budesonide, or placebo was administered to preterm infants <28 weeks' GA within 24 hours of life and continued until the infant was weaned off respiratory support or until 32 weeks' PMA (whichever was earlier). The budesonide group had a significantly lower incidence of BPD compared to the control group (27.8% vs. 38%; *p* = 0.004); however, the mortality rate was higher, albeit not significant (16.9% vs. 13.6%, *p* = 0.17). Inhaled budesonide did not increase the risk of NDI (102).

In a small RCT consisting of 18 preterm infants with established BPD, Yuskel et al. demonstrated decreased coughing and wheezing during infancy and improved functional residual capacity six weeks after initiating beclomethasone therapy (103). However, no reduction in invasive MV duration, supplemental oxygen use, or respiratory symptoms was noted with fluticasone use in preterm infants with moderate-severe BPD in two RCTs (104, 105). The Cochrane review studying the effect of late (>7 days) ICS use in preterm infants included eight studies and did not find a decline in BPD rate at 36 weeks' PMA or mortality. No increase in adverse events was seen with late ICS use (106).

A Cochrane review by Shah et al. found ICS and systemic PNS to be similar in terms of incidence of BPD at 36 weeks' PMA, mortality, GI bleeding, NEC, ROP, or neurodevelopment (107). Intratracheal administration of corticosteroids with surfactant is discussed in the surfactant section of this review.

*Early inhaled budesonide or early systemic dexamethasone use cannot be recommended due to the increased risk of GI perforation, NDI, and mortality. Late (>7 days) PNS may be considered in selective infants dependent on invasive MV and/or high concentrations of supplemental oxygen if the benefits of PNS use are likely to be higher than the risk of adverse effects according to the clinical judgment of the provider; these infants are at high risk of mortality or adverse neurodevelopmental outcomes. The risk of BPD for infants* <*31 weeks' GA can be calculated using the neonatal BPD outcome estimator available at* *https://neonatal.rti.org/index.cfm* *and may be used to assess the risks and benefits of PNS in these infants (**108**). The neonatal provider should explain these risks and benefits to the patients' parents and the decision regarding PNS use should be made jointly with them. When systemic PNS use is considered, the highest benefit is seen when PNS are administered between 8-49 days of life* (89).

### Prophylactic Non-steroidal Anti-inflammatory Drugs (NSAIDs)

Gay et al. first suggested that in mechanically ventilated preterm infants, a large PDA with a left to right shunt for more than four days was associated with the development of severe BPD (109). A hemodynamically significant PDA results in increased pulmonary fluid filtration rate, reactive inflammatory changes, and alteration in the maturation of the pulmonary vasculature, all of which contribute to the pathogenesis of BPD (110).

A multicenter RCT consisting of 421 patients with hemodynamically significant PDA showed a 79% closure rate in infants treated with indomethacin at <5 days of age, compared to a 28% closure rate in infants receiving placebo (*p* < 0.05). However, this increase in the PDA closure rate did not correlate with a decrease in BPD or mortality in the indomethacin group (111). Although prophylactic NSAIDs have been shown to increase the rate of PDA closure in preterm infants, the utility of prophylactic indomethacin or ibuprofen in reducing morbidity and mortality has remained controversial. A Cochrane review published in 2010 did not find a difference in the rate of BPD or duration of oxygen use with prophylactic indomethacin administration (112). In a large retrospective study by the NICHD research network consisting of 7,831 infants, prophylactic indomethacin use was associated with a significantly lower requirement for PDA treatment after the first day of life, without a decrease in the incidence of BPD, death, or their combined outcome (113).

In a prospective double cohort-controlled study consisting of 397 infants, Liebowitz and Clyman showed a lower risk of BPD and that of BPD or death when indomethacin treatment was started <15 h of age compared to conservative treatment (114). On the contrary, Pan et al. showed a higher incidence of BPD in infants receiving prophylactic indomethacin (55 vs. 41%, *p* = 0.014) (115). The recent Cochrane review by Ohlsson et al. included only one RCT comparing the incidence of BPD at 36 weeks' PMA and that of mortality with prophylactic (<72 h) treatment of asymptomatic PDA with IV ibuprofen to placebo (116). Prophylactic ibuprofen did not decrease the incidence of death or BPD (116, 117). The Baby-OSCAR trial (ISRCTN84264977) and the BeNeDuctus trial (NCT02884219) are multicenter RCTs designed to study the difference between prophylactic (<72 hours) treatment with ibuprofen vs. placebo. These studies will likely provide more information and help build a consensus about prophylactic NSAIDs use (118, 119).

Indomethacin treatment in neonates can cause oliguria and acute kidney injury. An increased risk of gastrointestinal perforation has been observed with simultaneous use of corticosteroids and NSAIDs (112, 116, 120).

*In the absence of evidence of improved BPD outcomes, we do not recommend routine prophylactic NSAID therapy for BPD prevention in preterm infants*.

### Vitamin A

Retinol or Vitamin A is required for the growth and differentiation of respiratory epithelial cells. Plasma vitamin A and retinol-binding protein levels were low in preterm infants who developed BPD (121). Vitamin A for BPD prevention was initially studied in 1987 in an RCT by Shenai et al. 40 infants at high risk of developing BPD were enrolled. Infants in the Vitamin A group received 2000 IU of intramuscular Vitamin A every other day starting postnatal day 4 for a total of 14 doses. The incidence of BPD declined remarkably with Vitamin A treatment (45 vs. 85%) (122). This trial, however, was conducted before the widespread use of surfactant therapy and antenatal corticosteroids.

An RCT by Pearson et al. conducted in 1992, consisting of 49 infants using the same dose as Shenai et al., did not reveal a benefit in decreasing BPD with Vitamin A use (46 vs 44%) (123). Due to inconsistent results obtained with various studies regarding the benefit of retinol in the prevention of BPD, a study was conducted by the NICHD neonatal research network to determine the appropriate dosage of Vitamin A that could be administered without significant side effects. Vitamin A administered at 5,000 IU/dose 3x/week for 12 doses was found to be the most effective in increasing serum retinol levels in preterm infants without signs of clinical toxicity (124). A follow-up RCT published by the NICHD neonatal research network in 1999 used this dose and demonstrated a decrease in CLD rate with vitamin A use (number needed to treat (NNT) = 14–15) (125). Subsequently, most studies used a dose of 5,000 IU or higher.

In animals subjected to oxidative lung injury, retinoic acid supplementation has been shown to increase mean alveolar area, improve alveolar regeneration, decrease pulmonary fibrosis, and prevent oxidative damage to the diaphragm (126–128).

The Cochrane review demonstrated a minimal risk reduction in BPD at 36 weeks' PMA and the composite outcome of death or oxygen requirement at four weeks of life with Vitamin A administration. The combined risk of death or BPD at 36 weeks' PMA was not altered with Vitamin A use (129). A meta-analysis by Ding et al. (130) included nine RCTs and 1409 premature infants. This study showed a lower incidence of BPD after Vitamin A administration (OR: 0.67; 95% CI: 0.52–0.88). Vitamin A did not increase the incidence of ROP, NEC, IVH, sepsis, or mortality (130). Although Vitamin A has shown to have some effect in preventing BPD, the increased cost of therapy, pain caused due to intramuscular injections and the modest benefit of the treatment made the neonatologists question “is Vitamin A worth the shot?” (131).

Interestingly, a recent reanalysis of the NICHD RCT conducted in 1997 showed that the risk reduction of BPD or death was greater in infants with lower risk compared to those with higher risk after intramuscular vitamin A administration (125, 131).

Alternate routes for vitamin A administration are being explored. Two RCTs showed higher plasma retinol levels in preterm infants receiving 5,000 IU/day oral vitamin A for 28 days, albeit with no effect on reduction in BPD in either study (132, 133). Another RCT by Basu et al. demonstrated a reduction in the combined effect of death or BPD at 36 weeks' PMA (RR 0.444; 95% CI: 0.229–0.844) after oral administration of alternate day 10,000 IU/dose for 28 days (134). Further studies are necessary to determine the appropriate oral dose required for BPD prevention. A large phase-III RCT, the NeoVitA trial, is currently enrolling patients. This study will test the effect of high dose oral vitamin A administration (5,000 IU/kg/day × 28 days) on BPD and/or mortality at 36 weeks' PMA (135).

Inhalational or endotracheal vitamin A has also been shown to be effective in animal studies but has not yet been evaluated in human trials (136, 137).

*Based on current evidence, in centers with low rates of BPD, IM vitamin A cannot be recommended due to the minimal (if any) benefit and the associated discomfort*.

### Diuretics

Although regularly used in the NICU for BPD, diuretics remain a topic of controversy amongst neonatologists. Brief use of furosemide in BPD management has been reported as early as 1978. In a prospective study involving 28 preterm infants requiring MV, Sniderman et al. showed that the respiratory status improved in 20 of the 28 infants during the first trial and 14 of the 28 infants during the second trial of one week of furosemide therapy (138). Studies have shown improved short-term pulmonary compliance and decreased pulmonary resistance one to two hours after furosemide administration (139, 140). The decrease in alveolar-arterial oxygen gradient observed in the treatment group by Najak et al. was not observed by Patel et al. (141). However, the treatment group in the study by Patel et al. differed from that of Najak et al. in that none of the subjects in the former study were mechanically ventilated at the time of the study, and not all of them were diuretics-naïve (140, 141).

Only two RCTs consisting of a total of 39 infants have studied the long-term use (>1 week) of furosemide (142, 143). Improvement in oxygenation and compliance was noted in preterm infants > 3 weeks after 7–8 days of systemic furosemide use (142–144). Due to the potential complications of long-term furosemide, including nephrocalcinosis, hypokalemia, and hypochloremic metabolic alkalosis, neonatologists sought other diuretics for long-term use in infants with BPD.

In a randomized, double-blind crossover study by Kao et al., the use of chlorothiazide and spironolactone for one week was associated with a decreased mean airway resistance and increased mean dynamic lung compliance (145). However, in another parallel non-crossover RCT consisting of twenty-one infants with BPD, the combination of spironolactone-hydrochlorothiazide for one week did not affect the lung mechanics despite an increase in diuresis in the treatment group (146). In a subsequent placebo-controlled randomized trial by Kao et al., the chlorothiazide-spironolactone combination therapy was associated with a significant improvement in lung compliance (46%, *p* < 0.001) and resistance (36%, *p* < 0.05), and less supplemental oxygen use (p<0.01), when measured four weeks after the initiation of therapy. These favorable effects, however, did not persist after discontinuation of the diuretic therapy, and the use of diuretics did not decrease the number of days infants required supplemental oxygen (147).

A Cochrane review showed that airway resistance decreased significantly after one week of thiazide-spironolactone use but not after four weeks. The improvement of compliance was seen after one week and after four weeks of treatment. However, the authors advised exercising caution while interpreting the results due to the small number of patients included in this review (148).

The improvement of pulmonary function after diuretic use is independent of its effect on diuresis (140, 143, 146). As the adverse effects of furosemide use were associated with its action on the renal tubules, the idea that inhaled furosemide may be used to manage BPD was explored. Rastogi et al. first used nebulized furosemide in increasing doses in eight preterm infants with BPD dependent on MV (149). Lung compliance, pulmonary resistance, and tidal volume improved significantly 30 min to 4 h after a 1 mg/kg dose of nebulized furosemide without associated diuresis and renal adverse effects (149). Prabhu et al. showed no difference in the improvement of lung compliance and tidal volume in 13 preterm infants 24–28 weeks' GA dependent of MV and treated with 2 mg/kg compared to 1 mg/kg nebulized furosemide at >14 days of age (150). A Cochrane review including eight studies concluded that at a dose of 1 mg/kg, transient pulmonary function improvement was noted with aerosolized furosemide when used in preterm infants > 3 weeks of age with CLD (151).

None of the RCTs have demonstrated an effect on the duration of hospital stay, oxygen use or MV, or the incidence of BPD with the use of diuretics. However, a recent retrospective study from the Pediatrix medical group showed that more days of furosemide exposure (between postnatal day seven and corrected GA 36 weeks) was associated with a lower incidence of BPD and that of BPD/death (152).

The use of diuretics in neonatal ICUs varies widely throughout the country (153–155). In a retrospective study of over a hundred thousand infants < 32 weeks' GA and <1,500 g birthweight admitted to the NICUs managed by the Pediatrix group, 37% of infants received at least one diuretic, with furosemide being the most used (154). In another retrospective study including 41 hospitals with 1429 infants <29 weeks' GA diagnosed with BPD at 28 days of age, 86% infants received diuretics, and 4–86% infants received a diuretic course of > 5 days in the study hospitals (155). In 58% of all infants with established BPD and discharged home on diuretics, the diuretics were tapered or discontinued at the first outpatient visit. Also, the duration of outpatient therapy and taper were longer in patients discharged home on oxygen (156).

*Due to the side effects of diuretics use and the absence of benefits, we cannot recommend routine chronic use of diuretics. However, sporadic doses of furosemide may be considered in preterm infants with worsening respiratory status with chest radiograph showing evidence of pulmonary edema, based on clinical judgment*.

### Bronchodilators

Bronchodilators include beta-agonists [isoproterenol, albuterol (also known as salbutamol), levalbuterol and terbutaline], anticholinergics (atropine and ipratropium), and methylxanthines (theophylline, aminophylline, and caffeine). Lungs of infants with BPD often have increased resistance and decreased compliance (157). The use of beta-agonists has been shown to reduce airway resistance in both intubated and non-intubated BPD patients and improve dynamic compliance and specific airway conductance in intubated BPD patients (158–161). The use of salbutamol was shown to be effective in improving compliance and resistance in very low birth weight (VLBW) infants as early as the second week of life (162). Similarly, inhaled atropine has been shown to improve dynamic compliance in non-intubated patients with BPD (159).

Studies have shown variable results with the combined use of anticholinergic and beta-agonist agents. In a double-blind, placebo-controlled randomized trial performed by Kao et al., although metaproterenol and atropine improved airway resistance in infants with BPD, no synergy was noted with their combined use in these patients (161). However, another RCT by Brundage et al. noted a significantly decreased airway resistance and improved lung compliance one to four hours after treatment with salbutamol + ipratropium compared to ipratropium alone (163).

Inhaled beta-adrenergic agents cause bronchodilatation through their action on β-2 receptors leading to smooth muscle relaxation. Ipratropium and atropine antagonize the action of acetylcholine, thus preventing parasympathetic bronchoconstriction and decreasing secretions. However, despite the short-term improvement in pulmonary mechanics, the use of bronchodilators has not been shown to prevent, treat or reduce the severity of BPD (164–168).

To date, only one RCT has studied the efficacy of beta-adrenergic medications for the prevention of BPD. This study, including 173 infants <31 weeks' GA and randomized to four groups (placebo, salbutamol, beclomethasone or salbutamol and beclomethasone), did not show any improvement in either survival, BPD and severity of BPD or the duration of MV and oxygen therapy in any of the treatment groups (164). *Post-hoc* analysis from the NEuroSIS trial showed no benefit of early bronchodilators (<48 h of life) in decreasing the incidence of BPD and/or death (169). Nevertheless, bronchodilators, particularly albuterol, continue to be frequently used in the NICU for patients with BPD (170). This is due to the belief that a subset of patients demonstrates airway reactivity that does respond to bronchodilator treatment (171, 172).

In a retrospective study consisting of 40 patients <1500 g birthweight, Morrow et al. found that 21patients (52.5%) had a decrease in resistance of ≥10% on pulmonary function tests (PFTs) (performed at mean PMA 34.9 weeks) after bronchodilator use (172). Another prospective study consisting of 44 children with BPD who received PFTs at 6, 12, and 24 months after initial discharge from the NICU showed that only 30% of the patients responded to bronchodilator treatment (171). The use of bronchodilators in the NICUs is variable and ranged from 0-81% in one study that included infants <29 weeks' GA and <1500 g birth weight (164). Among infants with BPD at 28 days of life, the use of inhaled bronchodilators ranged from 0-59%. Tracheostomy, duration of respiratory support, and exposure to steroids and diuretics positively correlated with the use of inhaled bronchodilators (173).

Although various delivery methods have been used for the administration of inhaled bronchodilators, the use of a metered-dose inhaler (MDI) and a spacer is preferred over nebulizer due to the shorter time required to deliver the medication without causing cooling of gases and lower risk of paradoxical increase in airway resistance (174–176).

Adverse effects of beta-agonists reported to date include hypokalemia, tachycardia, arrhythmias, tremors, hypertension, and hyperglycemia while those of anticholinergics include tachycardia, decreased gastrointestinal motility, tremors, and dry airway (167). It is important to note that the beta-agonists when used in the presence of tracheomalacia, which is often present in infants with BPD, can cause worsening of wheezing due to smooth muscle relaxation of an already floppy airway (177).

Theophylline and aminophylline have been shown to decrease bronchospasm and improve compliance by their action on the adenosine receptors. However, they have a narrow therapeutic window, and the adverse effects include gastrointestinal symptoms, tachycardia, agitation, and hypertension (167). Consequently, their use in the NICU has declined markedly over the years (170).

*Thus, based on the current evidence, generalized use of bronchodilators cannot be recommended for either prevention or treatment of BPD. Larger RCTs studying the efficacy of bronchodilators in BPD are needed. Inhaled bronchodilators may be trialed for patients that demonstrate signs of increased airway resistance. Pulmonary function tests in infants with BPD may aid in selecting the appropriate candidates for the use of inhaled bronchodilators as well as their discontinuation when there is a lack of improvement*.

### Pulmonary Vasodilators

In preterm infants, pulmonary hypertension (PH) often complicates BPD and is known to increase mortality. PH in BPD has a fixed/structural and a reactive component. The fixed component is associated with abnormal development of the pulmonary vasculature following lung injury (178). The reactive component, associated with increased vascular resistance, is the target of various treatments used to treat BPD-associated PH (BPD-PH) (178). Endothelium-derived nitric oxide (NO) causes smooth muscle relaxation through increased levels of cyclic guanosine monophosphate (cGMP) (179). Impaired NO-cGMP signaling pathways have been implicated in BPD pathogenesis and BPD-PH development in experimental models (180). Pulmonary vasodilators such as inhaled NO, sildenafil and bosentan are often used in the NICU for management of BPD-PH.

#### Inhaled Nitric Oxide (iNO)

The use of iNO in preterm infants remains controversial. Endogenous NO promotes pulmonary vasodilatation by increasing cGMP and decreasing intracellular calcium (181). Through covalent attachment to cysteine, nitric oxide forms S-nitrosothiols, which improve peripheral vasodilatation (182). CLD models of preterm baboons were found to have lower nitric oxide synthase expression and enzyme activity and iNO treatment improved the ability of surfactant protein B to decrease surface tension, thus improving lung volume and dynamic compliance, and lowering expiratory resistance (183, 184). iNO has also been shown to reduce pulmonary inflammation and fibrin deposition, decrease endothelial cell apoptosis and enhance survival in rodent models of CLD (185, 186). It is known that early injury to the pulmonary vasculature results in smooth muscle proliferation, impaired vasodilatation, increased hypoxic vasoconstriction, and decreased arteriolar number, and pulmonary hypertension. Strategies that prevent pulmonary vascular injury may attenuate the development of PH associated with BPD (187).

Despite evidence supporting iNO use from pre-clinical studies, the benefits of iNO have not been proven in clinical studies involving preterm infants. iNO was first used in preterm infants with BPD who had acute hypoxemic respiratory failure due to pneumonia. At 3–10 ppm, iNO improved oxygenation in six infants with BPD complicated by pneumonia (188). Subsequent studies showed that iNO treatment also improved PaO2 in infants with severe BPD (189, 190). However, no evidence of PH was documented in either of these studies.

Unfortunately, there are no RCTs evaluating the efficacy of iNO in BPD-PH. iNO for the prevention of BPD has been studied in various RCTs. A Cochrane review studying the efficacy of iNO in preterm infants included seventeen RCTs (191). Compared to the control group, preterm infants who received routine iNO showed no difference in the overall incidence of death and/or BPD at 36 weeks' PMA (191–195). Similarly, no difference was noted in the above outcomes when the infants were treated with iNO based on supplemental oxygen requirement or BPD risk (191).

Another large RCT, the NEWNO trial published recently, consisting of 451 preterm infants <30 weeks GA and <1,250 g birthweight, receiving MV between postnatal age 5–14 days, showed no difference in BPD at 36 weeks' PMA or neurodevelopmental and respiratory outcomes at 18–24 months between the treatment and placebo groups (196).

In the NOCLD trial, the BPD-free survival was significantly higher with iNO treatment in African American infants compared to placebo (Relative benefit 1.66, 95% CI 1.16–2.37) (197). In the EUNO trial, although not significant, BPD-free survival was higher in black infants compared to non-black infants (194). However, in the study by Kinsella et al., no significant difference was reported in the composite outcome of death, intracranial hemorrhage, or periventricular leukomalacia between white and non-white infants ≤ 34 weeks gestation, treated with prophylactic iNO. This study did not report on the difference in BPD incidence between the two race groups (193). A recently published meta-analysis including three RCTs and a total of 1240 infants <34 weeks' GA found that black infants treated with iNO had a lower incidence of BPD at 36 weeks PMA and that of death or BPD at 36 weeks' PMA compared to those who received placebo (198).

iNO therapy appears to be mostly safe with no increase in the incidence of IVH, NDI, CP, or ROP when used in preterm infants (191). However, long-term data regarding iNO use in preterm infants are limited. Interestingly, follow-up at age one year of 456 infants enrolled in the NOCLD study showed a significantly lower use of bronchodilators, ICS, systemic PNS, diuretics, and supplemental oxygen post-NICU discharge in infants who received iNO (199). Follow-up at seven years of infants enrolled in the EUNO trial did not demonstrate a significant difference in the hospitalization rates or the use of respiratory medications between the two groups (200).

Per the NIH consensus statement from 2010, the available evidence does not support the use of iNO either as routine or as rescue therapy in premature infants <34 weeks' GA requiring respiratory support. Additionally, the group has stated that iNO has been inadequately studied in rare conditions like pulmonary hypertension or hypoplasia in infants <34 weeks' GA, and in such cases, its use should be after discussing the risks, benefits, and uncertainties with the families (201). The data from studies published since this statement are insufficient to recommend routine iNO use for BPD prevention. Further studies are needed to confirm and evaluate the basis for the racial disparity in the responsiveness to iNO and the outcomes. (191, 195, 196, 198).

*Based on the current evidence, we do not recommend routine use of iNO for either BPD prophylaxis or the treatment of BPD-associated PH. However, iNO use may be considered in individual cases of BPD during acute PH crises*.

#### Sildenafil

Sildenafil is a selective phosphodiesterase-5 (PDE-5) inhibitor that inhibits the degradation of cGMP, thereby prolonging the actions of cGMP, resulting in smooth muscle relaxation. The use of sildenafil in the NICUs has increased over the years.

In a retrospective study by Thompson et al., sildenafil was administered to 0.11% of infants discharged from the NICUs managed by the Pediatrix medical group over 16 years, with an increase from 0% in 2001 to 0.17% in 2016 (202). Sildenafil is FDA-approved for the treatment of PH in adults, but its use in children remains off-label. There is a paucity of evidence on the safety and efficacy of sildenafil in patients with BPD-PH. Hon et al. first reported using sildenafil for the treatment of BPD-PH in 2005 (203). Most of the current literature on sildenafil in established BPD includes individual case reports, case series, or retrospective studies.

A recent meta-analysis published in 2019, which consisted of five retrospective studies including 101 patients, showed that sildenafil use was associated with an improvement in the respiratory scores in 15% infants (95% CI: 0.0–30.4) within 2–7 days and >20% improvement in the estimated pulmonary arterial pressure within 1–6 months in 69.3% (95% CI: 56.8–81.8) preterm infants with BPD-PH, without any serious adverse events (204). It is, however, difficult to ascertain from this study, whether the improvement in pulmonary arterial pressures was the result of treatment with sildenafil or simply that of tincture of time.

Side effects of sildenafil reported in children include hypotension, tachycardia, priapism, and facial flushing (205). A recent phase 1 trial by Jackson et al. found more adverse effects with faster intravenous use compared to enteral use. Adverse effects listed in this study included hypotension, NEC, and cardiogenic shock, of which only hypotension was determined to be drug-related (206).

Larger prospective studies and RCTs are required to study the utility and safety of this drug in the neonatal population. The SILDI-SAFE trial is a multicenter, sequential dose-escalating, placebo-controlled, double-blinded randomized study expected to complete in December 2023 and will provide valuable information on the safety of sildenafil in preterm infants with severe BPD (207). However, since this study will include only patients with BPD without PH, the efficacy of sildenafil to treat already established BPD-PH cannot be evaluated in this study.

Prophylactic sildenafil in neonatal rats exposed to hyperoxia has been shown to improve alveolarization, promote angiogenesis, decrease lung inflammation and fibrin deposition, and thus decrease BPD via activation of hypoxia-inducible factor signaling pathway (208–210). However, RCTs studying the effect of prophylactic sildenafil in preterm infants did not demonstrate any significant impact on BPD or death (211, 212).

*Sildenafil may be used in select premature infants with BPD-associated PH after consultation with a pediatric cardiologist and a pediatric pulmonologist; however, the quality of evidence for its use remains low. There is no role for sildenafil in BPD prevention outside of research setting*.

#### Bosentan

An increase in endothelin-1 (ET-1) expression occurs in the vascular endothelial cells in PH (213). Bosentan, a non-selective competitive antagonist of ET-1 receptor, has been shown to reverse endothelin-mediated smooth muscle constriction, hypertrophy, and hyperplasia (178, 214, 215).

Although not approved by the FDA for children under three years of age, off-label bosentan has been reported to improve PH in infants with BPD, either alone or in combination with sildenafil, in various retrospective studies (216–218). In common practice, it is often used in severe BPD as second-line therapy after sildenafil. A dose of 1–2 mg/kg/dose twice daily is used.

Larger prospective studies are needed to study the safety and efficacy of bosentan in the treatment of BPD-associated PH. In the rodent model of hyperoxia-induced lung injury, bosentan treatment was found to have a protective effect with lower fibrosis and inflammation (219). There are no studies evaluating bosentan use in preterm infants at risk of developing BPD.

Potential adverse effects of bosentan include hepatotoxicity, fluid retention, and dosage-related decline in hematocrit and hemoglobin. Liver function tests should be measured before starting bosentan and monthly thereafter (220).

*With no documentation of efficacy and the risk of serious adverse effects, we cannot recommend routine bosentan use in infants with BPD-associated PH at this time. However, where the use of bosentan as a second-line treatment for BPD-PH is considered, it should be done so after discussion with the pediatric cardiologist and after discussing the potential risks with the parents/family*.

### Macrolides

Ureaplasma colonization has been associated with the development of BPD in various studies (221–223). In a meta-analysis by Wang et al., the risk of development of CLD was 1.72 (95% CI 1.5–1.96) times higher in colonized infants than uncolonized infants (221). The anti-microbial activity of macrolides against Ureaplasma and their anti-inflammatory properties led to research into the use of macrolides to prevent BPD, where inflammation is a key driver. Macrolides exert their anti-inflammatory action by inhibiting neutrophil chemotaxis, tumor necrosis factor (TNF)-α, interleukin (IL)-1, and IL-6 (224, 225).

Earlier studies in the 20th century and the early 21st century did not demonstrate a decrease in either the incidence or the severity of BPD in preterm infants with erythromycin use at various dosages (226–228). This was thought to be due to the failure of erythromycin to eliminate Ureaplasma colonization (228).

Ballard et al. first studied the role of azithromycin in preventing BPD in 43 extremely low birth weight (ELBW) premature infants. In this pilot, double blinded, RCT, infants <72 h of life were randomized to receive a placebo or azithromycin within 12 h of starting MV. The study group received azithromycin 10 mg/kg/day x7 days followed by 5 mg/kg/day until the infant was off MV or supplemental oxygen for a maximum of 6 weeks. Although the rates of mortality and BPD were similar in the two groups, a significantly lower proportion of infants who received azithromycin received PNS (31 vs. 62%; *p* = 0.05). The treatment group also had a significantly lower MV duration (median 13 days vs. 35 days, *p* = 0.02) (229). This was thought to be due to increased anti-inflammatory and anti-microbial activity of azithromycin against Ureaplasma compared to erythromycin (229, 230). However, in addition to the lack of beneficial effect on mortality or BPD, a larger study consisting of 220 infants <1,250 g by the same group did not demonstrate any benefit in decreasing either PNS use or duration of MV (231).

Subsequently, two RCTs have demonstrated a significant reduction in BPD incidence at 28 days with azithromycin use (232, 233). IV azithromycin (10 mg/kg/day for five days) administered within 12 h of initiation of MV and within 72 h of birth significantly decreased the levels of plasma IL-2 and IL-8 in VLBW infants (233). A meta-analysis by Nair et al. found that azithromycin significantly reduced the incidence of BPD at 36 weeks' PMA (RR 0.83, 95% CI 0.71–0.98) (234). However, a recent RCT published in 2020 that included infants between 24–28 weeks' GA showed no significant improvement in BPD at 36 weeks' PMA or death with azithromycin treatment regardless of colonization status (235).

Azithromycin has been well-tolerated in neonates in various observational and randomized studies (236, 237). Azithromycin causes less diarrhea and abdominal discomfort compared to erythromycin. However, similar to erythromycin, azithromycin is associated with an increase in the risk of infantile hypertrophic pyloric stenosis, especially when administered within 14 days of life. This is likely due to their agonistic effect on the gastric motilin receptors (236, 238). Arrhythmias were not reported with the use of azithromycin in neonates despite the reports of prolonged QTc and torsades de pointes in adults (236).

A large RCT, the AZTEC trial (ISRCTN11650227), aims to study the effect of a 10-day course of early IV azithromycin (20 mg/kg (days 1–3) followed by 10 mg/kg (days 4–10) administered within 72 h of birth to neonates <29 weeks' GA) at 36 weeks' PMA and is currently enrolling patients (239).

Only one study using clarithromycin to prevent BPD has been published to date. This RCT showed a significantly lower rate of BPD in infants with Ureaplasma urealyticum-positive cultures treated with clarithromycin compared to placebo (2.9 vs. 36.4%; *p* < 0.001) (240). However, this was an underpowered study and enrolled only 30% of the intended subjects.

*While azithromycin may help decrease the incidence of BPD, there is insufficient evidence regarding the dosage, duration, and timing of therapy. Due to the risk of side effects, we do not currently recommend routine prophylactic use of azithromycin for BPD prevention in premature infants*.

## Future Directions

Table 3 outlines the various experimental drugs currently being studied for the management of BPD.

**Table 3:** New experimental drugs for BPD.

**Experimental drug**	**Proposed mechanism of action as seen in animal models**
Stem cell therapy • MSC • MSC exosomes/extracellular vesicles • Endothelial progenitor cells • Human amnion epithelial cells	• Improved cell growth, cell proliferation, and angiogenesis. • ↓ inflammation/fibrosis • ↓ oxidative injury
Other anti-inflammatory agents • DHA • CC10/CC16 • IL1RA • Pentoxifylline	• ↓ inflammation
Antioxidants • Superoxide dismutase	• ↓ inflammation • ↓ oxidative injury • Preservation of pulmonary angiogenesis
Nutritional supplements • Inositol • L-Citrulline	• Inositol: ↑ production of phosphatidylcholine and phosphatidylinositol • L-Citrulline: ↑ production of nitric oxide via role on arginine/NO pathway
Hormones • Erythropoietin (EPO)	• Upregulation of epidermal growth factor-line domain 7 • ↓ alveolar simplification • ↓ fibrosis • Improved angiogenesis
Growth factors and RNAs • rhIGF1/rhIGFBP3 • HIF-α • VEGF • miRNA, lncRNA	• ↑ cellular proliferation • Regulation of alveolarization and angiogenesis • ↓ oxidative stress

*The above experimental drugs, although promising, lack evidence of safety and efficacy, and must be used in research settings only*.

### Stem Cell Therapy

Mesenchymal stromal cell (MSC) therapy is a relatively new treatment that may prove to be an essential weapon in the neonatal provider's arsenal against BPD. However, it currently remains to be in the experimental phase. MSCs can be derived from many sources, including embryonic tissue, umbilical cord/umbilical cord blood, placental tissue or adult bone marrow, liver, or fat (241, 242). MSCs facilitate healing by preferentially entering sites of injury, secreting factors that promote cell growth, cell proliferation, and angiogenesis. They also secrete chemokines that prevent oxidation and fibrosis. Additionally, MSCs inhibit inflammation by acting as immunomodulators and inhibiting the proliferation and the function of lymphocytes, monocytes, and macrophages (241). Several pre-clinical studies have demonstrated the benefit of MSCs in animal models of BPD, and earlier administration is associated with better outcomes (243, 244). A meta-analysis of these studies showed that MSCs improved alveolarization, decreased inflammation, decreased fibrosis, prevented pulmonary remodeling, improved pulmonary angiogenesis, and resulted in lower rates of pulmonary hypertension compared to controls (245). Co-administration of MSC + surfactant is associated with higher lung injury compared to MSC alone, likely due to surfactant-induced decrease in the viability of MSC (246).

A very limited number of clinical trials have studied the use of MSC in preterm infants with risk of or diagnosis of BPD. Chang et al. performed the first phase I study using human umbilical cord blood-derived MSC (hUC-MSC; PNEUMOSTEM®) in nine preterm infants (24-26 6/7 weeks' GA). An intratracheal dose of 1 x 10^7^ cells/kg and 2 x 10^7^ cells/kg was delivered to the first three and the last six patients, respectively (247). hUC-MSC administration was demonstrated to be safe and inflammatory cytokines were lower in infants after hUC-MSC administration. Also, BPD severity was lower in the HUC-MSC group than in the matched controls (247). The 2-year follow-up showed decreased rates of home oxygen use and neurodevelopmental disability, including CP in the transplant group compared to the infants who did not receive MSC (248). This group recently completed a double-blind RCT recruited 66 infants (23–28 weeks' GA) receiving MV at the time of enrollment, randomly allocated to receive intratracheal 1 x 10^7^ cells/kg hUC-MCS or saline between days 5–14. Although infants who received hUC-MSC had decreased intratracheal inflammatory markers, the rate of death or severe/moderate BPD was similar between the two groups. Of note, severe BPD was lower with hUC-MSC use in the 23–24-week group (249). Due to the small sample size in each of these studies, the results must be interpreted with caution. Another phase I study from the United States enrolled 12 infants (23–27 6/7 weeks' GA and 500–1,000 g birth weight). 10/12 patients in the treatment group had severe BPD at 36 weeks (250).

There are ongoing phase I and phase II studies in Spain, Korea, Vietnam, and China (241).

The MSC-BPD trial (NCT03601416) is a randomized dose-escalating open-label phase II trial from China which will include 72 infants <1 year of age with the diagnosis of BPD, randomly allocated to control group, low-dose IV hUC-MSC (2.5 × 10^6^ cells/kg,) and high dose IV hUC-MSC (5 x 10^6^ cells/kg) groups in the 1:1:1 ratio (251). This will be a vital trial to study the efficacy of MSC in already established BPD.

MSC-extracellular vesicles/exosomes have also shown promise in various animal studies by immunomodulating macrophages, decreasing lung inflammation and fibrosis, as well as restoring lung architecture in hyperoxia-induced BPD (252–255). The UNEX-42 trial (NCT03857841) was a phase 1 RCT studying the safety of MSC exosomes in infants <27 weeks. It enrolled three subjects but was discontinued due to a business decision (256). Other forms of stem cells that have been studied include endothelial progenitor cells (EPCs) and human amnion epithelial cells (hAECs) (257–259).

### Erythropoetin (EPO)

Recombinant human EPO (rhEPO) treatment in neonatal rats exposed to hyperoxia upregulated epidermal growth factor-line domain 7 and was associated with decreased alveolar simplification, improved angiogenesis and decreased fibrosis (260, 261). However, EPO administration in preterm lambs increased alveolar inflammation and exacerbated ventilator associated lung injury (262). In a multi-center prospective cohort study including 867 neonates <28 weeks' GA, neonates with higher blood EPO concentrations (concentrations in the 4th quartile) on day 14 of life had a higher incidence of moderate but not severe BPD (263).

While retrospective studies have demonstrated a potential benefit of erythropoietin in reducing BPD in preterm infants, a meta-analysis consisting of 17 RCTs showed no difference in the incidence of BPD between infants receiving EPO and placebo (264–266). The PENUT trial (Preterm Erythropoetin Neuroprotection trial) also showed no reduction in the incidence of BPD after high-dose EPO supplementation (267). EPO use did not increase the incidence of ROP, sepsis or NEC (266, 267).

Administration of EPO in combination with MSCs has also been shown to attenuate lung injury by promoting angiogenesis and decreasing fibrosis in murine hyperoxia induced lung injury models (268, 269). EPO in combination with MSCs has not yet been studied in humans.

### Docosahexaenoic Acid (DHA)

DHA is a long-chain polyunsaturated fatty acid with anti-inflammatory and antioxidant properties (270). In the DINO trial, a large RCT consisting of over 600 infants < 33 weeks' GA, Manley et al. documented decreased BPD incidence in male infants and infants <1.25 kg consuming maternal breastmilk when mothers consumed tuna oil capsules (3000 mg/day) that contained a high amount of DHA. No difference was seen in female infants or infants >1.25 kg (271). However, a recent study, the N3RO trial including over a thousand infants <29 weeks' GA showed a significantly higher rate of BPD at 36 weeks' PMA and of BPD or death before 36 weeks' PMA in infants receiving 60 mg/kg/day of enteral DHA (272). Similarly, the MOBYDIck trial included mothers who delivered infants at <29 weeks' GA randomized to receive 1.2 g/day DHA capsules vs placebo. The investigators noted no significant improvement in BPD-free survival rates in infants whose mothers received DHA capsules (273). Due to lack of sufficient data, DHA must only be used in research setting. Larger clinical studies are required to determine the appropriate dose as well as to assess the efficacy of DHA in preventing BPD in extremely preterm infants.

### Clara Cell Protein

Clara cell kDa 10-16 protein (also known as CC10/CC16/uteroglobin) is an anti-inflammatory immunomodulator present in abundance in the bronchoalveolar lavage (BAL) fluid of neonates (274, 275). CC10 has been identified as a potential biomarker for BPD (276). Studies have shown contradicting results on whether CC10 is elevated or decreased in the cord blood of infants that develop BPD (277–279). However, the CC10 levels in BAL fluid were found to be lower in infants that developed BPD (280, 281). In an RCT consisting of 22 infants randomized to receive either placebo, 1.5 mg/kg of recombinant human CC10 (rhCC10) or 5 mg/kg rhCC10 intratracheally, rhCC10 was found to be safe and decreased IL-6, neutrophil count, and total protein concentration in the tracheal aspirate during the first 3 days of life, but no difference was noted between the three groups in terms of BPD at 36 weeks' PMA (282). In another multicenter RCT consisting of 88 infants 24 0/7 to 29 0/7 weeks' GA, a single dose of intratracheal rhCC10 (1.5 mg/kg or 5 mg/kg) was found to be ineffective in changing long term respiratory outcomes at 12 months of age (283).

### Superoxide Dismutase (SOD)

SODs are antioxidant enzymes shown to be decreased in various animal models of BPD (284, 285). Additionally, pulmonary angiogenesis was preserved after exposure to hyperoxia in transgenic mice that had increased expression of extracellular SOD (286). Rosenfeld et al. first showed a decrease in both radiologic and clinical features of BPD after subcutaneous administration of multiple doses of bovine SOD to infants with severe RDS (287). However, the RCT by Davis et al. found no difference in rate of death or that of BPD at 28 days or at 36 weeks' PMA in infants receiving recombinant human CuZn-SOD (rh-CuZn-SOD) or placebo. Of note, infants treated with rhCuZn-SOD had fewer episodes of wheezing, required asthma medications less often and had lower hospitalization rates before one-year corrected age (288). A recent study found no correlation between EC-SOD in serum and the risk of BPD in infants < 32 weeks' GA (289). SODs may potentially improve the long-term pulmonary outcomes of infants with BPD; however, further research is needed regarding their efficacy, safety and dosing before they can be used in the clinical setting.

### Pentoxifylline

Pentoxifylline, a synthetic methylxanthine derivative, is a non-selective phosphodiesterase inhibitor with anti-inflammatory properties (290). Additionally, pentoxifylline has also been shown to prevent fibrin deposition and improve lung vascularization in rat pups exposed to hyperoxia (291, 292). Lauterbach et al. demonstrated an improvement in lung resistance and compliance and a decreased oxygen requirement after treatment with nebulized pentoxifylline in five preterm infants with BPD (293). Subsequently, in a non-blinded RCT, Lauterbach et al. demonstrated a significant decrease in the risk of BPD after treatment with pentoxifylline compared to placebo (OR: 0.32; CI: 0.11–0.94) (294). However, a recent double-blind RCT consisting of 81 infants between 23 0/7 to 27 6/8 weeks' GA did not show a difference in oxygen supplementation at 40 weeks' PMA after treatment with 10 days of nebulized pentoxifylline vs placebo (290). While promising, there currently exist limited data on both the efficacy as well as safety and dosing of pentoxifylline for prevention and treatment of BPD.

### Citrulline

L-citrulline is byproduct of conversion of L-arginine and oxygen to NO (295). In the kidney, L-citrulline is converted back to L-arginine. As L-arginine has a low oral bioavailability, L-citrulline supplementation has been studied for its effect on the arginine/NO metabolism pathway, which has been shown to contribute to the development of BPD (295, 296). Hyperoxia exposure in rat pups was associated with lower L-citrulline and L-arginine concentrations in the plasma. L-citrulline administration in these pups exposed to hyperoxia resulted in preservation of lung angiogenesis and prevention of alveolar simplification and PH (297). Montgomery et al. showed that infants with BPD-associated PH had lower concentrations of L-citrulline (5–28 μmol/L) compared to those with BPD without PH (21–57 μmol/L) (*p* = 0.005) (298). On the contrary, infants with BPD had a higher concentration of citrulline in the tracheal aspirate compared to those without BPD. The authors theorized that this was likely due to an effort by the infants with BPD to prevent PH onset by producing higher NO levels (299). Lauterbach et al. described a case of successful treatment of BPD-associated PH with oral citrulline therapy (150 mg/kg/day x 70 days) in an ex-25 weeker male infant (300). L-citrulline may serve as a potential therapy for BPD-PH. However, in the absence of data from clinical studies regarding the efficacy and safety, the use of L-citrulline for BPD-associated PH should be reserved for research settings only.

### Inositol

Inositol is a nutrient that promotes the production of phosphatidylcholine and phosphatidylinositol, and preterm infants with a premature decline in myoinositol have a more severe course of RDS (301). Many RCTs have evaluated the role of inositol in prevention of RDS and BPD. While no acute adverse effects were noted with inositol use, a recent Cochrane review found that inositol supplementation did not decrease the rates of BPD, death and the composite outcome of BPD or death in preterm infants and recommended against further clinical trials in neonates (302, 303).

### Other Potential Treatments

Various potential targets including growth factors, anti-inflammatory agents, and antioxidants are currently being evaluated for the prevention of BPD in extremely premature infants. In a phase II RCT that included infants 23 0/7 to 27 6/7 weeks' GA, Ley et al. showed a significant decrease in severe BPD after treatment with recombinant human insulin-like growth factor (rhIGF-1) complexed with rhIGF-binding protein 3 (rhIGFBP3) (304). Other factors showing promise in the pre-clinical phase include growth factors such as HIF-1α and VEGF, anti-inflammatory agents like interleukin-1 receptor antagonists (ILR1A), and long non-coding and micro RNAs (lncRNA and miRNA) (305–310).

## Conclusion

BPD continues to be one of the leading causes of mortality and morbidity in extremely preterm infants. Most of the pharmacologic agents currently in use for either the prevention or management of BPD provide minimal benefit, if any. While surfactant therapy and antenatal corticosteroids have failed to prevent BPD, one cannot underestimate their role in preventing RDS and overall mortality in preterm infants. Of the drugs currently available, early caffeine therapy provides the most promise in preventing BPD without increased risk of adverse effects. At our institution, we regularly prescribe caffeine to all infants <30 weeks' GA at birth, and it is continued until 34 weeks' PMA.

Due to the lack of evidence regarding the use of the other medications in BPD, we currently do not have any guidelines/protocols for their use. When using these medications, the neonatal provider should carefully weigh their possible benefits against the myriad of side effects most of them cause and individualize the therapy based on the infant's clinical picture. At this time, routine use of these medications cannot be recommended. Newer experimental therapies appear to be promising for BPD treatment/prevention in extremely preterm infants; however, further research is necessary before their safety and efficacy in clinical practice can be established.

## Author Contributions

RS conceptualized the study and drafted the manuscript. RD conceptualized the study and revised the manuscript for critically important content. RS and RD reviewed the final draft of the manuscript and approved the final version. Both authors contributed to the article and approved the submitted version.

## Conflict of Interest

The authors declare that the research was conducted in the absence of any commercial or financial relationships that could be construed as a potential conflict of interest.

## Publisher's Note

All claims expressed in this article are solely those of the authors and do not necessarily represent those of their affiliated organizations, or those of the publisher, the editors and the reviewers. Any product that may be evaluated in this article, or claim that may be made by its manufacturer, is not guaranteed or endorsed by the publisher.
